# A Digital Lock-In Amplifier for Use at Temperatures of up to 200 °C

**DOI:** 10.3390/s16111899

**Published:** 2016-11-11

**Authors:** Jingjing Cheng, Yingjun Xu, Lei Wu, Guangwei Wang

**Affiliations:** 1 School of Automation, Huazhong University of Science and Technology, Wuhan 430074, China; chengjj@hust.edu.cn (J.C.); xyjdyx643@hust.edu.cn (Y.X.); 2 School of Chemical Engineering and Environment, Beijing Institute of Technology, Beijing 100000, China; 3 Well-Tech R&D Institutes, China Oilfield Service Limited, Beijing 101149, China; wanggw4@cosl.com.cn

**Keywords:** digital lock-in amplifier, high temperature, SiP, MCM, thermal resistance, LUT method

## Abstract

Weak voltage signals cannot be reliably measured using currently available logging tools when these tools are subject to high-temperature (up to 200 °C) environments for prolonged periods. In this paper, we present a digital lock-in amplifier (DLIA) capable of operating at temperatures of up to 200 °C. The DLIA contains a low-noise instrument amplifier and signal acquisition and the corresponding signal processing electronics. The high-temperature stability of the DLIA is achieved by designing system-in-package (SiP) and multi-chip module (MCM) components with low thermal resistances. An effective look-up-table (LUT) method was developed for the lock-in amplifier algorithm, to decrease the complexity of the calculations and generate less heat than the traditional way. The performance of the design was tested by determining the linearity, gain, *Q* value, and frequency characteristic of the DLIA between 25 and 200 °C. The maximal nonlinear error in the linearity of the DLIA working at 200 °C was about 1.736% when the equivalent input was a sine wave signal with an amplitude of between 94.8 and 1896.0 nV and a frequency of 800 kHz. The tests showed that the DLIA proposed could work effectively in high-temperature environments up to 200 °C.

## 1. Introduction

A lock-in amplifier is an extremely effective system for detecting a weak signal with a low signal-to-noise ratio [1,2,3,4]. It has been widely used in various kinds of instrument, as well in logging tools, such as nuclear magnetic resonance (NMR) logging tools, acoustic logging tools and resistivity logging tools [5,6,7,8]. The exploration of petroleum resources requires drilling at depths of up to about 5000 m [9], where the ambient temperature can reach up to 200 °C. Logging tools suffer high failure rates at such depths, mainly because the performance of the components is constrained by the temperature [10,11]. Only a small number of conventional electronic components can reliably operate above 150 °C. Passive cooling methods, such as placing the instrument in a Dewar flask [12,13], are traditionally used to improve the operabilities and reliabilities of downhole tools in high-temperature/pressure environments, but such methods generally provide cooling only for a short time because of limited heat absorption. Logging-while-drilling tools are required to work continuously for not less than 100 h [14], so such tools reach excessively high temperatures if passive cooling methods are used. Therefore, lock-in-amplifiers used in logging-while-drilling tools must be designed with care, and the individual components of these lock-in amplifiers need to be able to withstand temperatures up to 200 °C. In this paper, we proposed a high temperature (200 °C) DLIA equipped with a packaging technology which significantly reduces system thermal resistance and improves the temperature performance. Experiments showed that the proposed DLIA could continuously work for 4 h while maintaining good performance.

## 2. System Architecture

**Figure 1:**
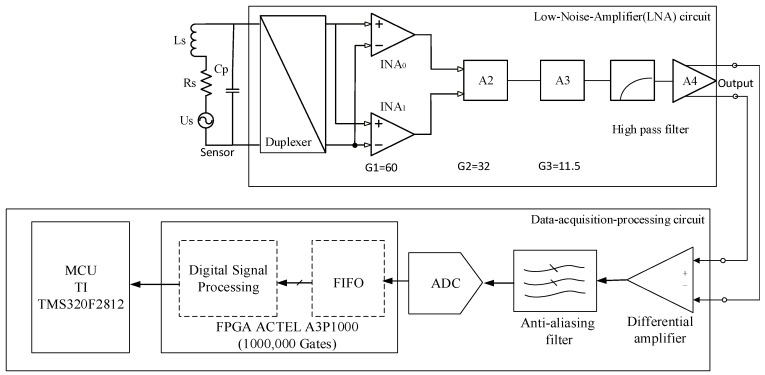
Structures of the lock-in amplifier circuit, the acquisition circuit, and the processing circuit.

We designed a digital lock-in amplifier (DLIA) with the structure shown in Figure 1 to acquire weak signals (~100 nV) in high-temperature environments. The main components of the DLIA are a low-noise amplifier (LNA) and a data acquisition and processing circuit. The signal is transmitted in differential mode between the LNA circuit and the data acquisition and processing circuit to improve the capacity of resisting disturbance.

The LNA consists of three parts, a gain amplifier, a high-pass filter (HPF), and a single-ended-to-differential converter. The gain amplifier amplifies the raw signal from the sensor to bring it to within an appropriate range. The HPF removes low-frequency noise, and differential transmission is achieved using the single-ended-to-differential converter.

**Figure 2:**
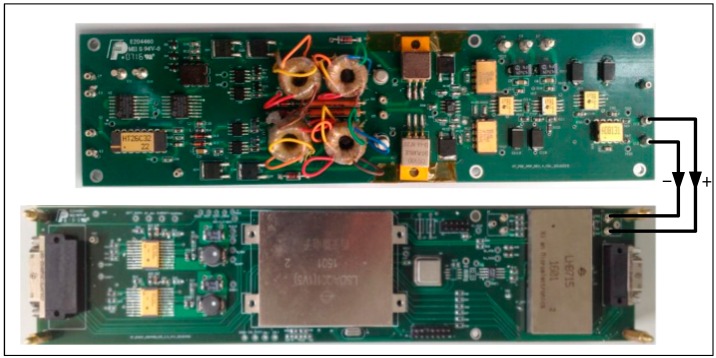
Photograph of the lock-in amplifier circuit, acquisition circuit, and processing circuit.

The data-acquisition-processing circuit contains a differential receiver, a band-pass filter (BPF), an analogue-to-digital converter (ADC), a field-programmable gate array (FPGA), and a microcontroller unit (MCU). The differential signal from the LNA is converted into a single-ended signal, then passed through the BPF before being transmitted to the ADC. The FPGA reads the digital data from the ADC and performs the DLIA algorithm, then the signal amplitude and phase are calculated by the MCU. The actual DLIA circuit is shown in Figure 2.

## 3. High Temperature DLIA

### 3.1. Low-Noise Amplifier

**Figure 3:**
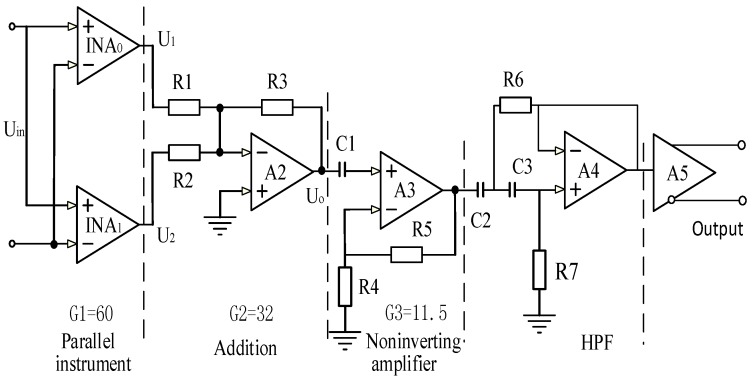
Schematic of the low-noise amplifier.

The basic LNA structure consists of a three-stage cascade. The circuit is shown schematically in Figure 3. The input stage is performed by two parallel instrument amplifiers. The outputs of the amplifiers are further amplified by parts of the operational amplifier circuit (A2, A3, in Figure 3), then the signal is passed through a second-order HPF to cut off the DC signal and low-frequency part. The signal finally passes through a transmitter to convert the single-ended signal into two differential signals.

According to the formula for the addition circuit, $U_{0}$ can be represented as shown in Equation (1):

(1) $$U_{0}=-R_{f}(\frac{U_{1}}{R_{1}}+\frac{U_{2}}{R_{2}})$$

where *U*_1_ and *U*_2_ are the outputs of the parallel instrument amplifiers, as shown in Equations (2) and (3), respectively:

(2) $$U_{1}=K_{1}U_{1in}+U_{1en}$$

(3) $$U_{2}=K_{2}U_{2in}+U_{2en}$$

In Equations (2) and (3), *K*_1_ and *K*_2_ are the magnifications, *U*_1*en*_ and *U*_2*in*_ are the output noise voltages, and *U*_1*in*_ and *U*_2*en*_ are the input voltages of the two instrument amplifiers, which are parallel and symmetrical. Thus ideally:

(4) $$\left\{ \begin{array}{l} K_{1}=K_{2}=K\\ U_{1in}=U_{2in}=U_{in}\\ R_{1}=R_{2}=R \end{array}\right.$$

Therefore, Equation (1) can be rewritten as:

(5) $$U_{0}=-R_{F}(\frac{U_{1}}{R_{1}}+\frac{U_{2}}{R_{2}})=-2R_{F}(\frac{KU_{in}+(U_{1en}+U_{2en})/2}{R})$$

in which *U*_1*en*_ and *U*_2*en*_ are unrelated random noise. The accumulative average is an effective method for filtering random noise, thus:

(6) $$\frac{KU_{in}}{(U_{1en}+U_{2en})/2}>\frac{KU_{in}}{U_{1en}}$$

Furthermore, the parallel instrument amplifiers can also effectively decrease the random noise level. In this design, there are only two instrument amplifiers in parallel because the size of the circuit board is limited.

All the components were selected with some care. C1, C2, and C3 are capacitors of Negative Positive Zero (NP0) material, which is a kind of Multilayer Ceramic Chip (MLCC) capacitor from the TDK Corporation (Peachtree City, GA, USA) and the temperature coefficient of capacitance is ±30 ppm/°C, for filtering and decoupling the capacitance. The temperature coefficient of the capacitance is extremely stable, so the value remains constant at high temperatures. R6 and R7 are thin film resistors. High-temperature tantalum power filter capacitors were selected. An AD8229 (Analog Devices, Norwood, MA, USA) was chosen for use as the instrument amplifier. This is a low-noise instrument amplifier with a noise voltage spectral density of $\ln V/\sqrt{Hz}$ and an operating temperature up to about 210 °C. An OPA211 amplifier (Texas Instruments, Dallas, TX, USA) was selected for use in the operational amplifier circuit and HPF circuit because of its high precision, allowing its use up to 210 °C. Good performance at high temperature required special attention to be paid to the layout of the key signal, which needed to be as short as possible to avoid affecting the magnification. We manufactured the printed circuit board out of high-temperature polyimide.

**Figure 4:**
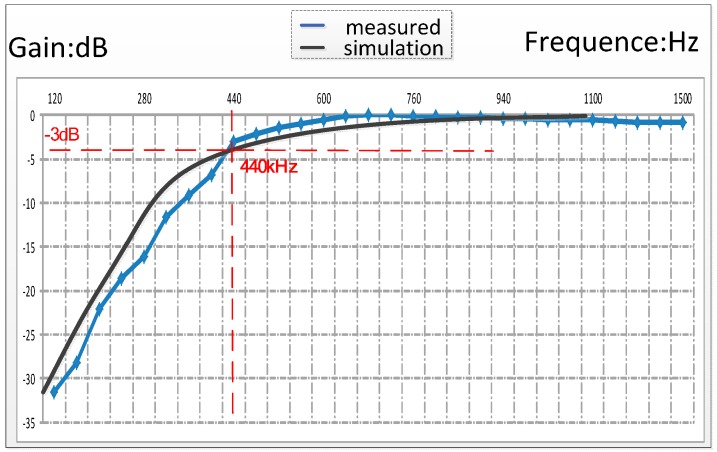
The waveform of simulation and experiment results about the HPF frequency characteristics.

In most well-logging applications, the signal frequency is between 500 kHz to 1000 kHz. So we designed the cut-off frequency of the HPF in LNA as 440 kHz. Both simulation and experiment results of the HPF frequency characteristic are shown in Figure 4. The black curve represents the software simulation diagram, and the blue curve represents the measured waveform.

### 3.2. High-Speed Data Acquisition System-in-Package

**Figure 5:**
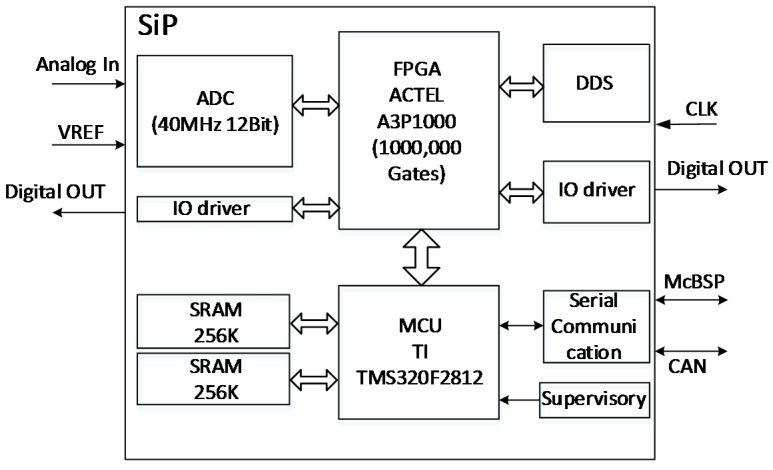
Components of the SiP.

The internal structure of the data acquisition and processing system-in-package (SiP) is shown in Figure 5. A 12-bit ADC, with the maximal sampling rate at 40 MHz, converts the high-frequency input signal with a maximum of 4 Vpp into digital data. The FPGA controls the ADC timing and reads the result into the built-in FIFO (First In First Out). The digital signal is then processed in parallel, then the calculated results are sent to the MCU (Microcontroller Unit).

**Figure 6:**
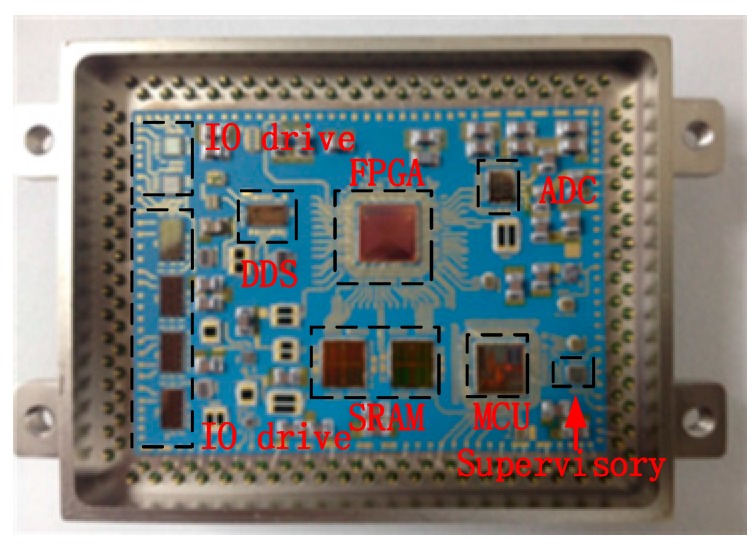
Internal structure of the SiP.

A picture of the SiP is shown in Figure 6. The MCU (TMS320F2812) and FPGA (A3P1000) chip wafers that could be used at high temperatures and the other Silicon-On-Insulator (SOI) chip wafers were provided by Xi’an Microelectronics (Xi’an, China). All the key passive components were suitable for use at high temperatures, with the material of NP0 texture capacitances and thin film resistors.

**Figure 7:**
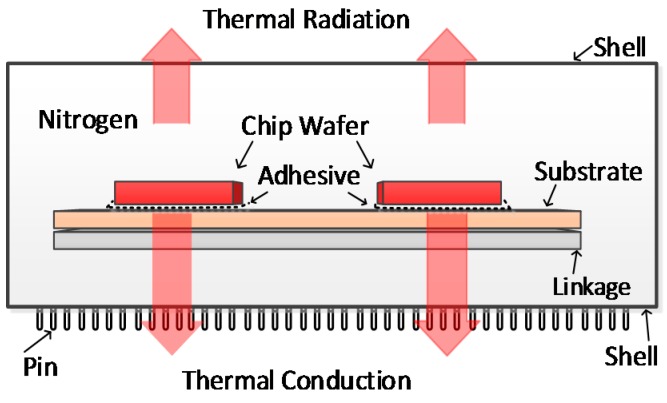
Equivalent heat structure chart for the SiP.

The equivalent heat structure chart for the SiP is shown in Figure 7. The wafers are bonded onto substrates with super-thin adhesive, and the substrate is connected to the linkage using co-fluxing alloy. All the materials are extremely thin, which helps decrease the thermal resistance of the system. The SiP needs to be filled with an inert gas (e.g., nitrogen) to prevent the wafers and other materials from becoming oxidized and corroded in the high-temperature environment. The low thermal conductivity of nitrogen means that heat in the SiP is mainly transferred from the substrate to the shell with rapidly dissipating to the external environment. The SiP shell acts as a cooling fin.

All the selected materials (substrates, linkages, and adhesives) were temperature resistant. The thermal conductivities [15,16] of the materials are shown in Table 1. Materials with higher thermal conductivities cause more rapid heat dissipation. The thermal expansion coefficients of the different materials need to be similar so that heating and cooling does not cause stress between the components, which can cause reliability problems and even damage to the chip wafer.

A thermal simulation model was established using COMSOL5.1 software, and the model was solved using the material parameters described above. Thermal resistance [17] can be described using Equation (7):

(7) $$R=\frac{d}{K\times A}$$

where *d* is the heat transfer distance (10 mm between the wafer and shell), A is the surface area of the wafer, and K=0.045 (W/mm)·℃ is the equivalent thermal conductivity coefficient.

The SiP equivalent thermal resistance is dominated by Equation (7), and the resistance of the FPGA is:

$$R_{FPGA}=\frac{10\;\mathrm{mm}}{0.045\;(℃/W)\times 10\;\mathrm{mm}\times 10\;\mathrm{mm}}=2.222\;\left(℃/W\right)$$

The equivalent thermal resistances of the other chip wafers were calculated using the same method, and the results are shown in Table 2. It can be seen from Table 2 that the equivalent thermal resistance of the system was decreased greatly by using the SiP. Heat around the wafer could be rapidly transferred to the external environment, improving the temperature class of the SiP internal chip wafer.

### 3.3. Signal Conditioning Multi-Chip Module

**Figure 8:**
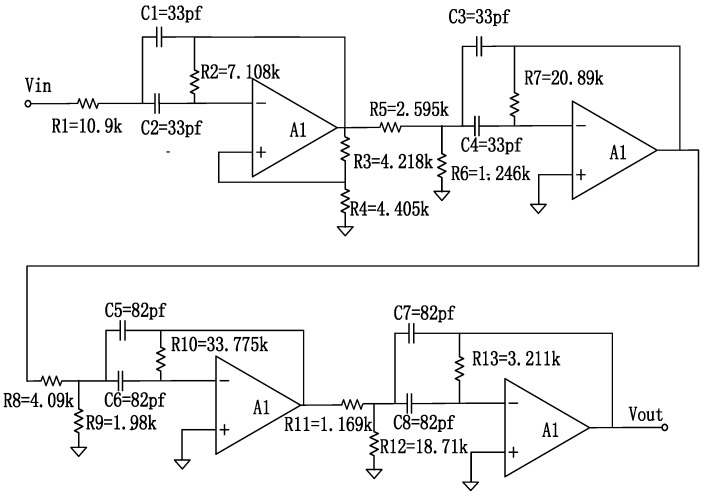
Schematic of the multi-chip module.

A schematic of the multi-chip module (MCM) signal conditioning process is shown in Figure 8. It mainly consists of an eight second order active BPF, which is implemented by the operational amplifier AD811. The AD811 is affected by low-temperature drift, the input offset voltage temperature drift being constant at 5 µV/°C.

**Figure 9:**
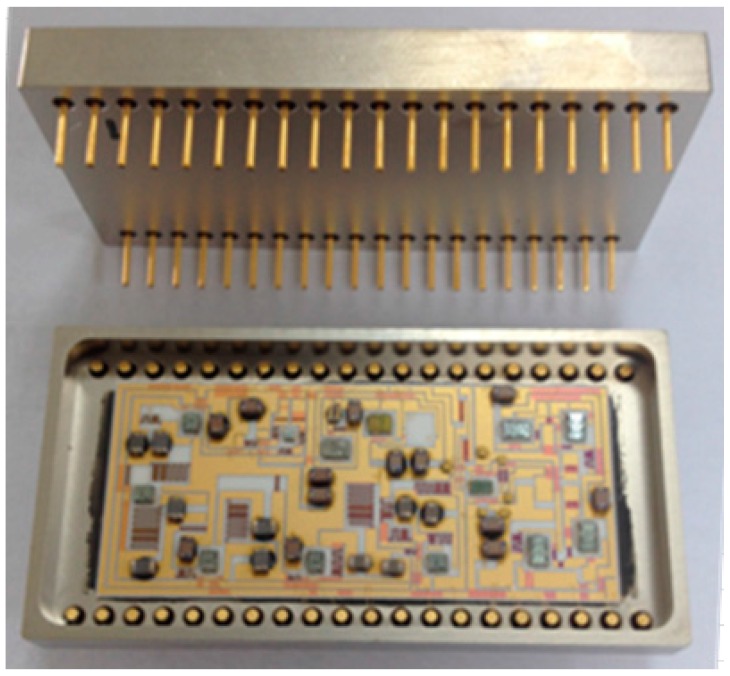
Photograph of the multi-chip module.

We used an MCM with a structure similar to that of the SiP to improve the AD811 temperature performance. All the passive components were selected for their performances at high temperatures. The MCM decreased the thermal resistance. As shown in Equation (7), the equivalent thermal resistance of the AD811 in the MCM was 19.753 **°**C/W, whereas the thermal resistance of the single chip was 155 **°**C/W. The MCM hardware is shown in Figure 9.

**Figure 10:**
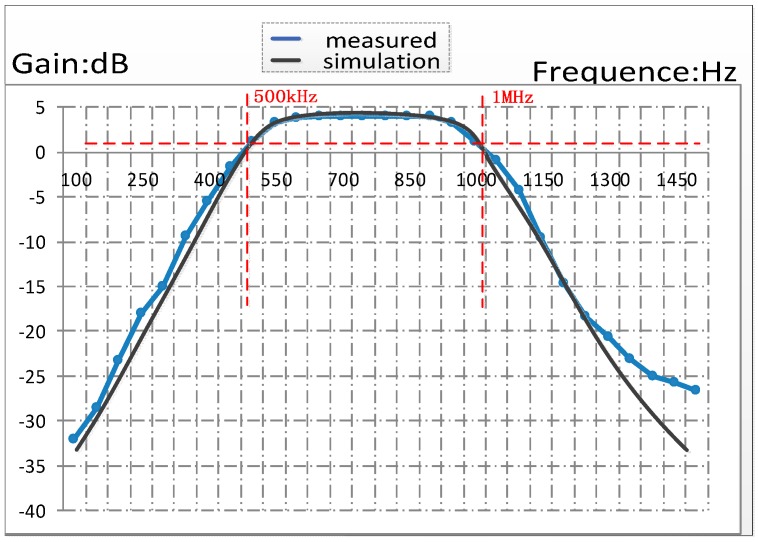
The waveforms of simulation and experiment results of the BPF frequency characteristics.

Both simulation and experiment results of the BPF frequency characteristics, performed using EDA software, are shown in Figure 10, and the black curve represents the software simulation diagram, and the blue curve represents the measured waveform. The results indicated that the gain was 4.211 dB, and the bandwidth was 500.5 kHz (within the pass-band, the lower limit cut-off frequency was 498.8 kHz and the upper limit cut-off frequency was 999.3 kHz).

### 3.4. Digital Lock-In Algorithm for a Low-Field Nuclear Magnetic Resonance Logging Application

There are many available methods to implement digital lock-in amplifier algorithms [18]. We used a digital lock-in amplifier algorithm in an FPGA of SiP that was similar to a low-field nuclear magnetic resonance (NMR) logging application [19]. An average filter was designed and added to the MCU to remove additional noise [20].

Essentially, the mixing calculation was a series of accumulating multiplications performed in the traditional way (multiplication then accumulation). A sine signal was used as the reference when the mixing calculations were performed. The output was:

(8) $$\begin{array}{l} I=\overset{M-1}{\underset{m=0}{\sum }}\overset{N-1}{\underset{n=0}{\sum }}s\left(mN+n\right)\sin\left(\frac{2\pi n}{N}\right)\\ \;=\overset{N-1}{\underset{n=0}{\sum }}\left[\overset{M-1}{\underset{m=0}{\sum }}X\left(n+mN\right)\right]\sin\left(\frac{2\pi n}{N}\right) \end{array}$$

where *N* is the sampling rate and *M* is the sampling period number. According to the distributive and associative laws, Equation (8) can be expressed as:

(9) $$\begin{array}{ll} I & =\overset{N-1}{\underset{n=0}{\sum }}\overset{M-1}{\underset{m=0}{\sum }}X\left(n+mN\right)\sin\left(\frac{2\pi n}{N}\right)\\ & =\overset{N-1}{\underset{n=0}{\sum }}Y\left(n\right)\sin\left(\frac{2\pi n}{N}\right) \end{array}$$

where $Y\left(n\right)=\;\sum_{m=0}^{M-1}X\left(n+mN\right)$ We performed the accumulation calculations first and the multiplications second to decrease the time required to perform the multiplications. *Y*(*n*) could be described using Equation (10):

(10) $$Y(n)=\sum_{b=0}^{B-1}Y_{b}[n]\times 2^{b},\;Y_{b}[n]\in [0,1]$$

where *Yb*[*n*] is the first b bit of *Y*(*n*). Therefore:

(11) $$I\;=\overset{N-1}{\underset{n=0}{\sum }}\sum_{b=0}^{B-1}Y_{b}[n]\times 2^{b}\sin\left(\frac{2\pi n}{N}\right)$$

According to the distributive and associative laws, Equation (11) can be rewritten as

(12) $$I=\sum_{b=0}^{B-1}2^{b}\times \sum_{n=0}^{N-1}Y_{b}[n]\sin\left(\frac{2\pi n}{N}\right)$$

The multiplication and accumulation calculations were conducted using a Look-Up-Table (LUT) in the FPGA, and the large table was split into multiple tables. Equation (12) could be expressed as Equation (13) when *N* = *L*_1_ + *L*_2_ +…+ *L_K_*:

(13) $$I=\sum_{b=0}^{B-1}2^{b}\times \left[\begin{array}{l} \sum_{n=0}^{L_{1}-1}Y_{b}[n]\sin\left(\frac{2\pi n}{N}\right)+\\ \sum_{n=L_{1}}^{L_{1}+L_{2}-1}Y_{b}[n]\sin\left(\frac{2\pi n}{N}\right)+\\ \cdots +\sum_{n=N-1-L_{K}}^{N-1}Y_{b}[n]\sin\left(\frac{2\pi n}{N}\right) \end{array}\right]$$

Equation (13) could also be expressed as:

(14) $$I=\sum_{b=0}^{B-1}2^{b}\times \left\{\begin{array}{l} T_{L1}\left[Y_{b}[0],\cdots ,Y_{b}[L_{1}-1]\right]+\\ T_{L2}\left[Y_{b}[L_{1}],\cdots ,Y_{b}[L_{1}+L_{2}-1]\right]+\\ \cdots +T_{LK}\left[Y_{b}[N-1-L_{K}],\cdots ,Y_{b}[N-1]\right] \end{array}\right\}$$

The LUT calculations using Equation (14) were conducted using Equation (15):

(15) $$\sum_{n=0}^{L_{1}-1}Y_{b}[n]\sin\left(\frac{2\pi n}{N}\right)$$

The convolution calculation was similar to the sine when the reference signal was cosine, the coefficient calculation process and the principle about the method of look-up table can refer to the reference [21].

**Figure 11:**
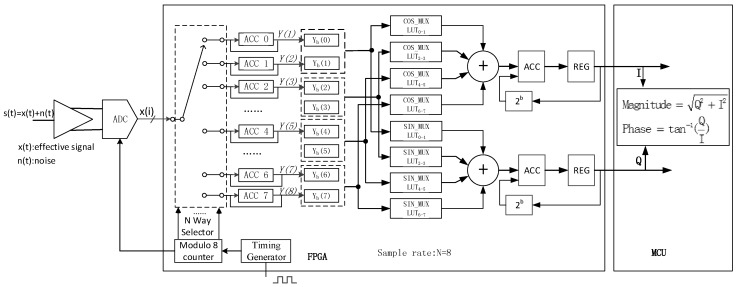
Implementation of the digital lock-in algorithm in the field-programmable gate array (FPGA) and microcontroller unit (MCU).

The implementation of the digital lock-in algorithm in the FPGA + MCU is shown in Figure 11. The eight-bit data were divided into four-bit data with highs and lows, and the four-bit data were further divided into two-bit data with highs and lows. The results of the reference sine multiplied by the low two-bit data from the low four-bit data were obtained using LUT^①^ in the Table 3, the results of the reference sine multiplied by the high two-bit data from the low four-bit data were obtained using LUT^②^, the results of reference sine multiplied by the low two-bit data from the high four-bit data were obtained using LUT^③^the results of the reference sine multiplied by the high two-bit data from the high four-bit data were obtained using LUT^④^, the results of the reference cosine multiplied by the low two-bit data from the low four-bit data were obtained using LUT^⑤^, the results of the reference cosine multiplied by the high two-bit data from the low four-bit data were obtained using LUT^⑥^, the results of the reference cosine multiplied by the low two-bit data from the high four-bit data were obtained using LUT^⑦^, the results of the reference cosine multiplied by the high two-bit data from the high four-bit data were obtained using LUT^⑧^, and the total length of the LUT was only 4 × 2^2^ = 16. Using the LUT method, which mainly adopted the RAM of the FPGA to fixed the accumulative coefficient, would turn the traditional method into a shifted accumulator. This avoided multiplication, occupied less resources, and greatly increased the data processing speed. The FPGA resource consumption using the traditional method and LUT method are compared in Table 4.

From Table 4 it is clear that the digital lock-in algorithm based on LUT implementation in a FPGA conserved more resources. Using the traditional method, a sampling rate of eight would require 41% of the logical units, whereas using the method of LUT would require only 13% of the logical units. Therefore, it was the ideal choice when need to improve the sampling rate. The traditional method commonly fails to meet the requirements of many instruments, in logging and aerospace for example.

## 4. Experimental and Results

**Figure 12:**
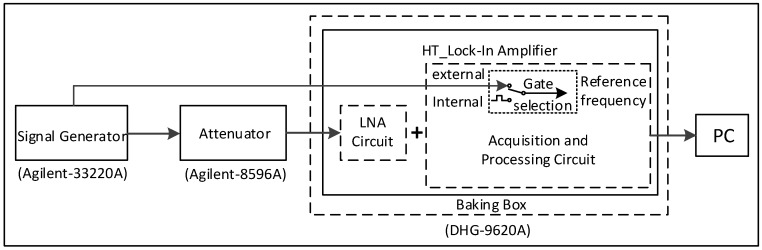
Schematic of the high-temperature digital lock-in amplifier test setup. The reference frequency of the digital lock-in amplifier was provided by an internal or external reference depending on the working method. The external reference provided by the signal generator (consistent with the frequency of the output signal) was used in the test process. The internal reference was controlled by the MCU, which could generate a clock signal with the requested frequency to act as the internal reference.

The experimental setup is shown schematically in Figure 12. The DLIA was placed in an oven (9620A; DHG, Shun-Nuo Instrument Technology, Tianjin, China), and a signal generator (33620A; Agilent Technologies, Santa Clara, CA, USA) generated a sine signal with different frequencies and peak–peak values. The signal was sent to the DLIA after being attenuated by 110 dB by an attenuator (8496A; Agilent Technologies, Santa Clara, CA, USA). The amplitude and phase of the signal were calculated and uploaded to the PC by the DLIA system through a serial port. The sampling rate *N* was eight and the period number of each sample *M* was 64. There were several choices for the DLIA reference frequency (i.e., the internal and external reference) depending on the different test mode.

During all our temperature experiment, According to the United States Department of Defense temperature test method standards [22], the rate of temperature rise was controlled at about 2 °C/min, and the temperature rise from 25 °C to 175 °C, then maintain 1 h. When temperature rising at 200 °C, it had been kept for 4 h. The total experimental time is about 8.6 h. The specific test performance indices and experimental data are shown below.

### 4.1. Linearity

**Figure 13:**
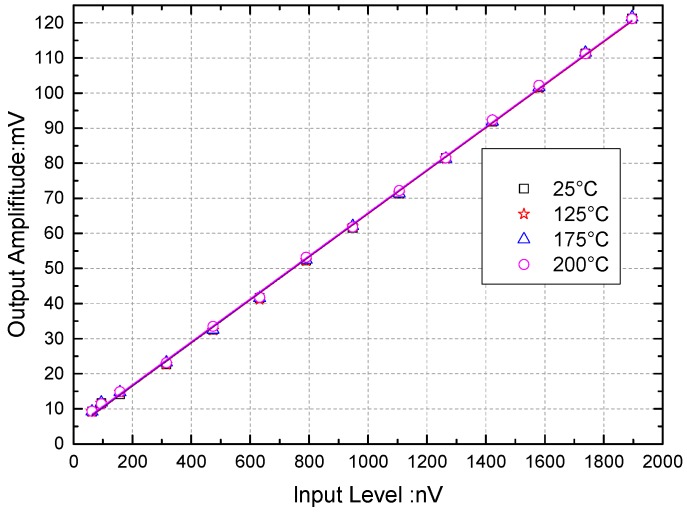
Outputs of the digital lock-in amplifier (DLIA) change along with the sine wave input from a variable amplifier. The DLIA input frequency was 800 kHz, and the equivalent amplitude range of the sine wave signal was 94.8–1896.0 nV. The theoretical gain of the DLIA was about 96.211 dB.

The generator exported a sine wave signal with a frequency of 800 kHz and an amplitude range of 30–600 mV to test the linearity of the DLIA. Before being sent to the DLIA, the signal was attenuated by 110 dB, which caused the input to have an equivalent amplitude of about 94.8–1896 nV. The internal reference was used as the DLIA reference signal (i.e., the DLIA reference signal had a fixed frequency of 800 kHz throughout the experiment). The linearity of the relationships between the DLIA inputs and outputs in different temperature at 25, 125, 175, and 200 °C are shown in Figure 13.

The non-linear errors and equivalent gains of the DLIA at different temperatures are shown in Table 5, in which represents the ratio between the maximum deviation of the measured value Δ*y_m_* and the measuring range *y_FS_*, and *ε =* Δ*y_m_*/*y_FS_* × 100%. As shown in Table 5, the nonlinear error increased from 1.488% to 1.736% between 25 and 200 °C.

### 4.2. Q Value

The *Q* value of the DLIA can be described using Equation (16):

(16) $$Q=\frac{f_{0}}{\Delta f}$$

where *f*_0_ is the centre frequency and Δ*f* is the DLIA bandwidth.

The generator exported a sine wave signal with a frequency of 750 kHz and an amplitude of 500 mV when the *Q* value was tested. An attenuation of 110 dB brought the input equivalent amplitude to 1580.0 nV. The internal reference was used as the DLIA reference signal, meaning that the DLIA reference signal was at a fixed frequency of 750 kHz throughout the test.

**Figure 14:**
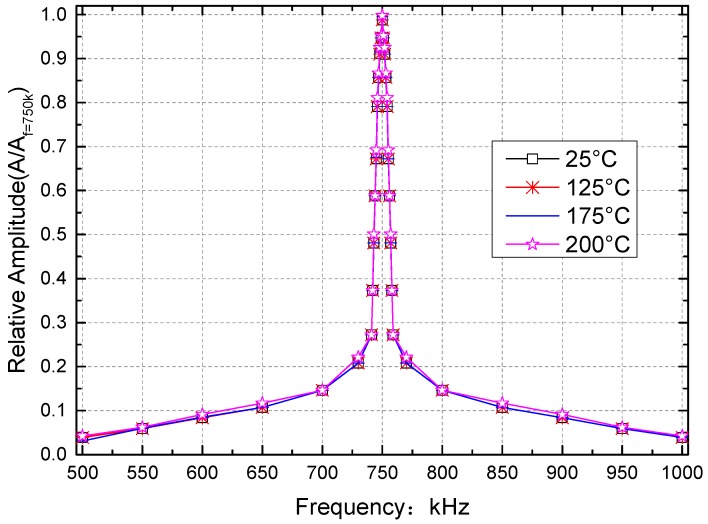
Experimental digital lock-in amplifier frequency responses. The signal generator output amplitude was 500 mV, and the frequency was 750 kHz. The signal was decreased by 110 dB using an attenuator. The theoretical gain of the digital lock-in amplifier was about 96.211 dB.

The experiments described below were used to calibrate the centre frequency and the *Q* value of the DLIA, and the results obtained at different temperatures are shown in Table 6. The lock-in-amplifier was equivalent to a BPF with a narrow bandwidth, and the equivalent BPFs of the DLIA at 25, 125, 175, and 200 °C are shown in Figure 14. The DLIA *Q* value was 1042 at 25–175 °C and 1041 at 200 °C.

### 4.3. Frequency Band Characteristic

**Figure 15:**
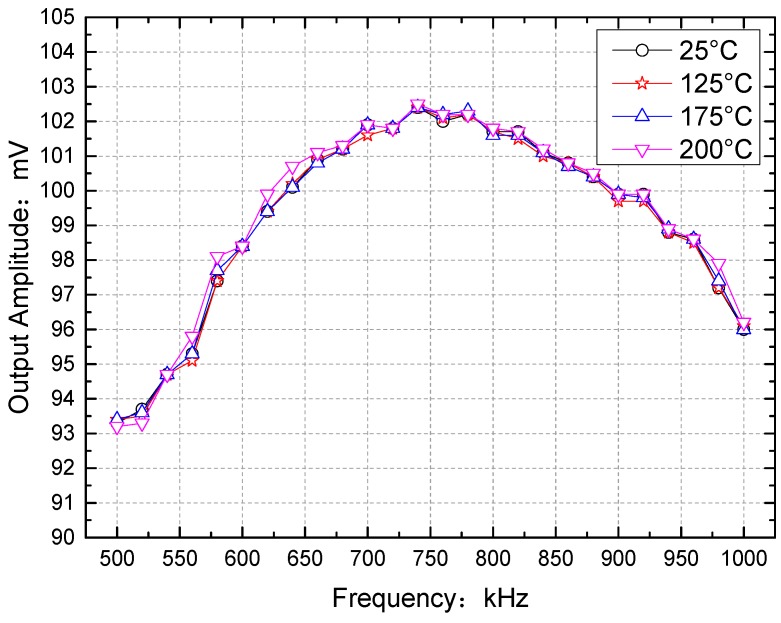
Relative amplitude plotted against the variable frequency of an input sine wave. The signal generator output amplitude was 500 mV, the frequency range was 500 kHz to 1 MHz, the step frequency was 20 kHz, and the attenuator decreased the signal by 110 dB. The theoretical gain of the digital lock-in amplifier was about 96.211 dB.

The generator exported a sine wave signal with an amplitude of 500 mV, a frequency range of 500 kHz to 1 MHz, and a step frequency of 20 kHz when the frequency band characteristics were tested. The other conditions were the same as in the earlier experiments. The frequency band characteristics of the DLIA between the inputs and outputs at 25, 125, 175, and 200 °C are shown in Figure 15.

The frequency band characteristics at different temperatures were studied by determining the average relative error ($E^{\prime }$) and the squared sum of the error (*E_esq_*).The average relative error $E^{\prime }$ was defined in Equation (17), which is the half valve of the difference between maximal and relative error. *X*(*t*) is the measured value and *S*(*t*) is the theoretical value. $E^{\prime }$ represents the difference between the measured and theoretical curves:

(17) $$E^{\prime }=\frac{1}{2}\times \left[\max\left(\frac{X\left(t\right)-S\left(t\right)}{S\left(t\right)}\right)-\min\left(\frac{X\left(t\right)-S\left(t\right)}{S\left(t\right)}\right)\right]$$

In Equation (17), *X*(*t*) is the measured value and *S*(*t*) is the theoretical value. The energy difference (sum of squared errors) between the measured value and the theoretical value can be described using Equation (18):

(18) $$E_{rsq}=\frac{\sum_{i=1}^{N}{\left[X\left(t\right)\right]}^{2}-\sum_{i=1}^{N}{\left[S\left(t\right)\right]}^{2}}{\sum_{i=1}^{N}{\left[S\left(t\right)\right]}^{2}}$$

where *X*(*t*) is the measured value and *S*(*t*) is the theoretical value.

The mean maximum relative errors and sum of squared errors at 125, 175, and 200 °C relative to 25 °C are shown in Table 7.

It can be seen that between 125 and 200 °C, the mean maximum relative error *E*’ was about 0.0010 and the sum of squared error *E_rsq_* was about 0.0039 relative to measurements at 25 °C. The experimental results show that The performance of our DLIA at high temperature is equivalent to the room temperature one in [23,24].

## 5. Conclusions

We have presented a DLIA consisting of a data acquisition processing system device SiP, an analogue signal conditioning device MCM, and the high temperature commercial devices AD8229 and OPA211. The DLIA can operate at environmental temperatures of up to 200 °C. The system can work at high temperatures because the individual components were designed and selected carefully. The DLIA was evaluated at high temperatures (up to 200 °C). Tests were performed at different temperatures, and the highest nonlinear error was about 1.736%. We found that the consistency of the DLIA’s frequency band characteristic was acceptable within the signal frequency range 500 kHz to 1 MHz. Experimental results showed that the DLIA is well suited to detecting weak signals in high-temperature environments, especially for well logging, aerospace exploring and many other high temperature and harsh environment fields.

## Figures and Tables

**Table 1:** SiP structural materials and characteristics.

Name	Material	Thermal Conductivity W/(mm·°C)
Substrate	PWB	0.013
Shell	Steel	0.012
Wafer	Si	0.161
Gas	Nitrogen	0.0228
Linkage	Eutectic	0.295
Bonder	Epoxy	0.002
Header	Kovar	0.017

**Table 2:** Thermal resistances between the single chips and the system-in-package.

	Single Chip	SiP
Name	Thermal Resistance (°C/W)	Equivalent Thermal Resistance (°C/W)
FPGA	26.1	2.222
MCU	35.0	3.472
ADC	100.0	24.691
DDS	87.0	23.641
SRAM	76.4	4.535

**Table 3:** The results of LUT about the different reference.

**sin_LUT^①^**		**sin_LUT^②^**		**sin_LUT^③^**		**sin_LUT^④^**	
Input	Output	Input	Output	Input	Output	Input	Output
00	0	00	0	00	0	00	0
01	0	01	256	01	0	01	7936
10	181	10	181	10	8011	10	8011
11	181	11	437	11	8011	11	7755
**cos_LUT^⑤^**		**cos_LUT^⑥^**		**cos_LUT^⑦^**		**cos_LUT^⑧^**	
Input	Output	Input	Output	Input	Output	Input	Output
00	0	00	0	00	0	00	0
01	256	01	0	01	7936	01	0
10	181	10	8011	10	8011	10	181
11	437	11	8011	11	7755	11	181

**Table 4:** Resource consumption using the field-programmable gate array (FPGA) in the traditional method and the look-up-table (LUT) method.

Implementation	Occupied Core Cells	Percentage (%)
Traditional way	10,076/24,576	41%
Method of LUT	3191/24,576	13%

**Table 5:** Relative nonlinear errors and equivalent gains of the digital lock-in amplifier at different temperatures.

Parameter	Temperature: *T* (°C)			
	25	125	175	200
*ε*	1.488%	1.488%	1.653%	1.736%
Equivalent Gain (dB)	96.210	96.210	96.187	96.311

**Table 6:** *Q* values for the digital lock-in amplifier at different temperatures.

Parameter	Temperature: *T* (°C)			
	25	125	175	200
*Q*	1042	1042	1042	1041

**Table 7:** Mean maximum relative errors and sum of squared errors relative to 25 °C.

Parameter	Temperature: *T* (°C)		
	125	175	200
*E*’	−0.00050	−0.00050	0.0010
$E_{rsq}$	−0.0011	0.0039	0.0037
